# Molecular dynamic simulations reveal structural insights into substrate and inhibitor binding modes and functionality of Ecto-Nucleoside Triphosphate Diphosphohydrolases

**DOI:** 10.1038/s41598-018-20971-4

**Published:** 2018-02-07

**Authors:** Jamshed Iqbal, Syed Jawad Ali Shah

**Affiliations:** Centre for Advanced Drug Research, COMSATS Institute of Information Technology, Abbottabad, 22060 Pakistan

## Abstract

Ecto-nucleotidase enzymes catalyze the hydrolysis of extracellular nucleotides to their respective nucleosides. Herein, we place the focus on the elucidation of structural features of the cell surface located ecto-nucleoside triphosphate diphosphohydrolases (E-NTPDase1-3 and 8). The physiological role of these isozymes is crucially important as they control purinergic signaling by modulating the extracellular availability of nucleotides. Since, crystal or NMR structure of the human isozymes are not available – structures have been obtained by homology modeling. Refinement of the homology models with poor stereo-chemical quality is of utmost importance in order to derive reliable structures for subsequent studies. Therefore, the resultant models obtained by homology modelling were refined by running molecular dynamic simulation. Binding mode analysis of standard substrates and of competitive inhibitor was conducted to highlight important regions of the active site involved in hydrolysis of the substrates and possible mechanism of inhibition.

## Introduction

Ectonucleotidases modulates the cell surface located nucleotides level by hydrolyzing them to their respective nucleosides. Of particular interest, cell surface levels of adenosine- and uridine- triphosphates and diphosphates (ATP, ADP, UTP and UDP) are of critical importance as they control several cellular responses via direct modulation of purinergic receptor signaling^1,2^. P2X receptor signaling gets activated by the presence of ATP and P2Y receptors responds to ATP, UTP, ADP, UDP, ITP and their sugars while adenosine activates P1 receptor signaling^3^.

An important class of ectonucleotidase includes nucleoside triphosphate diphosphohydrolases (NTPDases), formerly classified as ATPDases, E-type (extracellular) ATPases, ecto-ATPases or ecto-pyrases^4^. Eight different isozymes of NTPDases (namely, NTPDase1-8) have been identified among them NTPDase1-3 and 8 are cell surface located members and are typically known as E-NTPDases, other members are either intracellularly located and undergo secretion after heterologous expression as observed in case of NTPDase5 and 6 or are solely intracellularly located as in case of NTPDase4 and 7 isozyme^5^.

E-NTPDase1-3 and 8 consist of two membrane spanning domains with an active site facing extracellularly that catalyzes the hydrolysis of nucleoside triphosphates to their respective diphosphates which are subsequently hydrolyzed to their respective monophosphates^6,7^. Highly conserved regions of NTPDases are known as ‘apyrase conserved regions’ commonly abbreviated as ACR1-5^8–10^. These regions have been known to play a significant role in forming the catalytic site^11^. Deletion and mutations in amino sequence of ACR regions result in alterations of hydrolysis activity and substrate specificities^12–15^.

All E-NTPDase isozymes require divalent Mg^+2^ or Ca^+2^ ion in millimolar concentration for maximal hydrolysis activity. Absence of these divalent metal ions or their chelation by EDTA/EGTA renders these isozymes completely inactive^6,16^. The E-NTPDase isozymes differentially hydrolyze substrate molecules, E-NTPDase1 hydrolyzes adenosine triphosphate and diphosphate equally well. Thus, ATP is hydrolyzed almost directly to AMP with very less observable amount of ADP. Contrarily, UTP hydrolysis by ENTPDase-1 leads to accumulation of high amount of UDP^16^. E-NTPDase2 has a preference for hydrolysis of ATP over ADP up to 30 folds. Therefore, they hydrolyze ATP to ADP with higher rate as compared to the ADP hydrolysis into AMP leading to high levels of ADP accumulation^17^. The E-NTPDase3 is expected to effectively hydrolyze nucleoside triphosphate (up to 3 folds) but have a delayed hydrolyzing effect on nucleoside diphosphate^6^. However, E-NTPDase8 have been reported to show intermediate hydrolysis activity for nucleoside diphosphate and triphosphate^16^.

These enzymes are primarily expressed in leukocytes, endothelial cells and platelets, thus performing various biological processes such as hemostasis, vascular contraction, pain perception, vascular permeability, angiogenesis, inflammation and immune systems by regulation of the extracellular nucleotide levels^18^.

The E-NTPDase1 limits the intravascular platelet aggregation by hydrolyzing the aggregatory ADP to anti-aggregatory adenosine and is thus a novel therapeutic target for thrombotic diseases^19^. In contrast to the E-NTPDase1, the E-NTPDase2 on the other hand hydrolyzes ATP to ADP more rapidly as compared to ADP hydrolysis, ADP being agonist for platelet aggregation and thrombosis, therefore it promotes platelet aggregation and thrombosis^17^. Imbalanced ATP/ADP levels have been observed in aortic aneurysm and coronary artery diseases^18^.

The E-NTPDases also have a major role in insulin secretion. E-NTPDase1 has been found in acinar cells, blood vessels and blood capillaries of pancreatic islets^20^. Similarly, E-NTPDase2 has also been found in acinar cells and in cells surrounding blood capillaries. E-NTPDase3 has been located specifically inside Langerhans cells of the pancreas^20,21^. ATP has been known to increase insulin secretion from islets of Langerhans through activation of metabotropic P2 receptor as well as ionotropic receptors^22,23^ while adenosine has been known to activate purinergic P1 receptor signaling and inhibits the insulin release^24,25^. E-NTPDases thus inhibits insulin secretion by two ways, firstly by hydrolyzing ATP and secondly, by the production of adenosine^21^.

Inhibitors of E-NTPDases thus hold promising therapeutic value against disease conditions where extracellular nucleotides are involved. Several inhibitors have been reported previously that includes reactive blue 2, N^6^-methyl 2′-deoxyadenosine 3′, 5′-bisphosphate, suramin and their derivatives^26^. The non-hydrolyzable nucleotide analogues either have no or very little effect on P2 receptor signaling and act as competitive inhibitors with an estimated Ki values in lower micromolar ranges. Namely, ARL 67156 (6-*N*,*N*-diethyl-D-β,γ-dibromomethylene ATP), and 8-thiobutyladenosine-5′triphosphate are potent non-hydrolyzable analogues of nucleotides that inhibit the individual E-NTPDase isozymes with varying degree of inhibitory potentials^27–30^.

In order to develop more potent, selective and specific inhibitors of ENTPDases, several efforts have been made previously. Fragment based and structure based drug design offers a very useful method for developing new chemical entities with enhanced potency. Structure based drug design relies on the three dimensional structure of target bio-molecule. The crystal structures of rat E-NTPDase1 and 2 have been reported previously^31–34^. However, no human E-NTPDase crystal or NMR structures are yet reported. Comparative homology modelling offers simple and comparatively reliable method of modelling of these unknown protein structures. Modelling of protein structures that exhibits lower sequence identity with the available template structures often requires refinement because of the poor geometry. Refinement of these modelled protein structures can be accomplished by running their molecular dynamic simulations of few to hundreds of nanoseconds^35^. Binding mode analysis study has also been used extensively to determine the possible mechanism of binding offered by several substrates and inhibitors inside various enzymes and receptors^36–38^.

## Results and Discussion

### Homology Modelling

Homology models of surface located E-NTPDase1-3 and 8 were constructed using Molecular Operating Environment (MOE) 2014, 09^39^. Amino acid sequences of the target isozymes were fetched into the software and closely related template structures were identified. Rat origin E-NTPDase1 and 2 crystal structures were found to be the most top ranking template structures identified in terms of percentage similarity, identity and *E*-value. Rat origin E-NTPDase1 crystal structure (PDB ID 3ZX3) was found to be the most top ranking template structure which exhibited 74% sequence identity with human origin isozyme. Similarly, rat origin E-NTPDase2 (PDB ID 4BR5, 3CJ1 and 4CD3) were identified as top ranking template structures with percentage identity of about 83%. Unfortunately, for E-NTPDas-3 and 8 no crystal or NMR template structure with higher percentage similarity and identity were available. Rat origin E-NTPDase1 and 2 are the only available crystal structure with considerable higher sequence identity of 42% and 44% with human origin E-NTPDase3 and 8, respectively.

Ten homology models had been constructed for each E-NTPDase isozymes and the most top ranking homology model for each isozyme was further refined and energy minimized. Sequence alignment and superposition of the constructed homology models with their respective template structures exhibited Cα RMSD values between 0.5–1.5 Å. Higher the percentage similarity of template structure with target amino acid sequence lower the Cα RMSD observed values. (See Fig. S1 of SI for complete sequence alignment).

Stereochemical quality of the constructed homology models in terms of their Phi-Psi (Ramachandran) plots also revealed that higher the percentage identity of template structure with target amino acid sequence, higher would be the stereochemical quality of the homology models constructed and it will have lower number of residues with bad geometry (outlier region of Phi-Psi plot). Since, template structure of rat E-NTPDase2 had the highest percentage identity of 83% with human isozyme, it exhibited highest stereochemical quality with all residues in favored and allowed region. E-NTPDase1 had also been observed to exhibit considerably higher stereochemical quality with 99.5% residues in favored and allowed region and 0.5% residues lie in outlier region. In case of E-NTPDase3 and 8, several residues were found outside the favored and allowed region of Ramachandran plot. 2.8% residues of E-NTPDase3 and 1.9% residues of E-NTPDase8 were found to lie in outlier region of Ramachandran plot which necessitates refining of these structures. (See Fig. S2a and S2b of SI for Ramachandran plot analysis).

### Refinement of E-NTPDase Isozymes after refinement with MD simulation

Molecular dynamic simulation offers a strategy for refining protein structures with bad geometries and had been used for several protein structures previously^40,41^. The protein system had been solvated and 100 mM of divalent metal ions concentration was added because of the previously reported data on significantly enhanced activity of these isozymes in the presence of higher calcium or magnesium ion concentration^6^.

**Figure 1:**
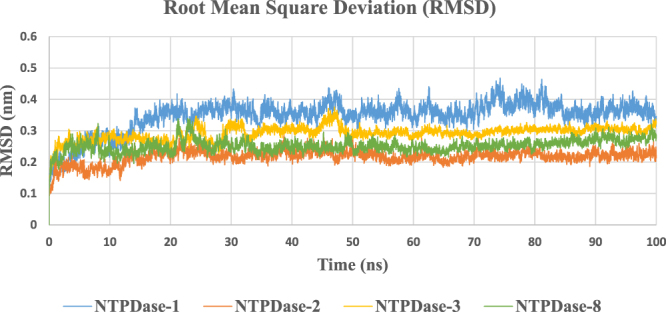
Root Mean Square Deviation (RMSD) of amino acid residues during simulation run.

Length of the simulation run was closely monitored and was terminated after confirmation of no further deviation in protein structure based on their RMSD values. Lowest deviation of about 0.2–0.25 nm from modelled structure was observed for E-NTPDase2, followed by E-NTPDase8 and 3. Highest deviation of up to 0.4 nm was observed in case of E-NTPDase1 isozyme. For all observed deviations, side chain deviations were more pronounced as compared to the residues backbone (data not shown). Figure 1 shows root mean square deviation of isozymes during 100 ns simulation.

### Protein Dynamics

**Figure 2:**
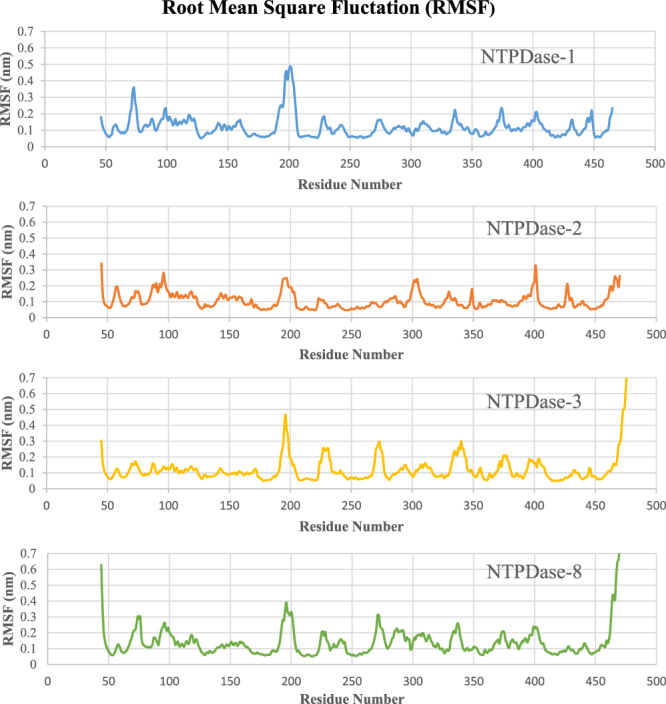
Root Mean Square Fluctuation (RMSF) of amino acid residues during 100 ns simulation run.

During simulation run the most pronounced root mean square fluctuation (RMSF) was observed for regions of highly non-conserved parts of the isozymes. The RMSF plot of each residue inside each isozyme can be found in Figure 2. Highest RMSF value was calculated for residues Gln191-Asn205 in case of E-NTPDase1, correspondingly residues with similarly high RMSF values includes Tyr183-Arg193, Glu199-Gly211 and Tyr186-Glu197 of E-NTPDase2, 3 and 8, respectively. All these residues belong to a loop part of protein further away from the active site region. Second region of high RMSF value includes residues Asn227-Asp234 of E-NTPDase1, the corresponding residues in case of E-NTPDase2, 3 and 8 were Ser215-Ala221, Glu233-Ser240 and Gly218-Ser225, respectively. E-NTPDase3 was found to fluctuate most amongst the second identified region of high RMSF values, which could be attributed to the fact that this region coils to α-helix during simulation run while no such recoiling was observed in case of other isozymes.

Another region of high RMSF values includes protein region formed by residues Ala63-Arg67 of NTPDase-1, Arg298-Cys310 and Cys399-Phe404 of E-NTPDase2, Val310-Val322 of E-NTPDase3 and His294-Asn304 of E-NTPDase8, respectively. Moreover, in case of E-NTPDase3 and 8 residues ranging from Gln277-Leu285 and Gln262-Arg270 respectively, had also been observed to have high RMSF values.

### Ramachandran plot analysis after Refinement

After running 100 ns of dynamic simulations, outliers in Ramachandran plot had been reduced significantly (see Fig. S2a and b for complete Ramachandran plots). For E-NTPDase2 no residue in outlier region was observed while in case of E-NTPDase1 and 8 only two residue lies in outlier region whereas for E-NTPDase3 isozyme, four residues were in outlier region. All E-NTPDase isozymes, number of residues present in additionally allowed region of Ramachandran plot declined while number of residues present in favored region rises significantly and is thus a clear evidence of improved stereochemical quality. Complete details of Ramachandran plot analysis is given under Table 1.

**Table 1:** Ramachandran Plot Analysis of E-NTPDase Isozymes.

E-NTPDase	Pre-refined Homology Models			Post-refined Homology Models		
	Favored Region	Allowed Region	Outliers	Favored Region	Allowed Region	Outliers
1	90.4% (378)	9.1% (38)	0.5% (2)	91.4% (381)	8.2% (34)	0.5% (2)
2	92.5% (392)	7.5% (32)	0.0% (0)	95.7% (403)	4.3% (18)	0.0% (0)
3	88.0% (375)	10.1% (43)	1.9% (8)	94.0% (406)	5.1% (22)	0.9% (4)
8	83.3% (354)	13.9% (59)	2.8% (12)	92.2% (392)	7.3% (31)	0.5% (2)

*(residue number).

### Isozymes Structures and Active Site Analysis

Different protein properties were calculated by using MOE software. E-NTPDase1 was found to be more compact and spherical in shape with lesser percentage helicity as compared to other isozymes. E-NTPDase1, 3 and 8 were found to resemble each other more closely in certain properties such as their isoelectric pH, net negative charge and zeta potential. Conversely for E-NTPDase2 relative to other isozymes, it has a more alkaline isoelectric point with net positive charge and zeta potential. Detailed protein properties are given in Table 2.

**Table 2:** Properties of E-NTPDase isozymes.

	E-NTPDase Isozymes			
	1	2	3	8
Protein mass (kDa)	47.98	46.48	48.75	46.87
Extinction coefficient (280 nm)	76375	62965	80385	86915
Percent helicity	30.9	33.9	33.6	34.8
Radius of gyration (Å)	23.65	23.10	23.55	23.64
Eccentricity	0.64	0.57	0.59	0.53
Van der Waal Surface area (Å^2^)	20236.1	18473.7	19999.2	20276.8
Hydrophobic surface area (Å^2^)	9832.6	10066.4	10419.3	10766.8
Hydrophilic surface area (Å^2^)	9314.5	7554.5	8627.1	8476.3
Isoelectric point (pI)	5.38	8.18	5.17	4.89
Average net charge (Z)	−9.16	1.22	−10.03	−11.74
Zeta potential (mV)	−31.54	9.76	−34.57	−40.47

Site finder of MOE software was used to probe possible binding sites based on size (i.e., on number of α-spheres included in a specific site), propensity of ligand binding (PLB) score^42^ for residues included, number of hydrophobic and side chain contact atoms present in that specific site. In E-NTPDase1, seven different sites with positive PLB values had been determined. For E-NTPDase2, 8 isozymes, eight sites with positive PLB values were found. While in E-NTPDase3, twelve possible binding sites with positive PLB values were observed. For each isozyme the top ranking largest site was considered for binding studies. Detailed dimensions of binding sites selected for further studies are given in Table 3.

**Table 3:** Top ranking binding site dimensions determined by Site Finder.

E-NTPDase Isozyme	Size	PLB	Hydrogen contacts	Side chain contacts	Hydrophilic surface area (A^2^)	Hydrophobic surface area (A^2^)	Van der Waal surface area (A^2^)
1	214	3.83	57	106	2262.8	2324.8	5235.3
2	283	4.50	66	146	2548.0	2267.0	5681.9
3	396	5.04	85	175	2766.2	3258.0	6920.8
8	326	5.26	80	168	2718.6	2511.0	6037.9

Sequence and structural alignment of the refined human E-NTPDase isozymes showed that E-NTPDase1 and 8 isozymes showed considerable high sequence similarity and identity with each other while E-NTPDase2 and 3 resembles each other more in terms of sequence identity and similarity as compared to other isozymes. ENTPDase-1 has a sequence identity of about 45% and sequence similarity of 62% with E-NTPDase8, while E-NTPDase2 and 3 have sequence identity of 43% and sequence similarity of about 62% with each other.

**Figure 3:**
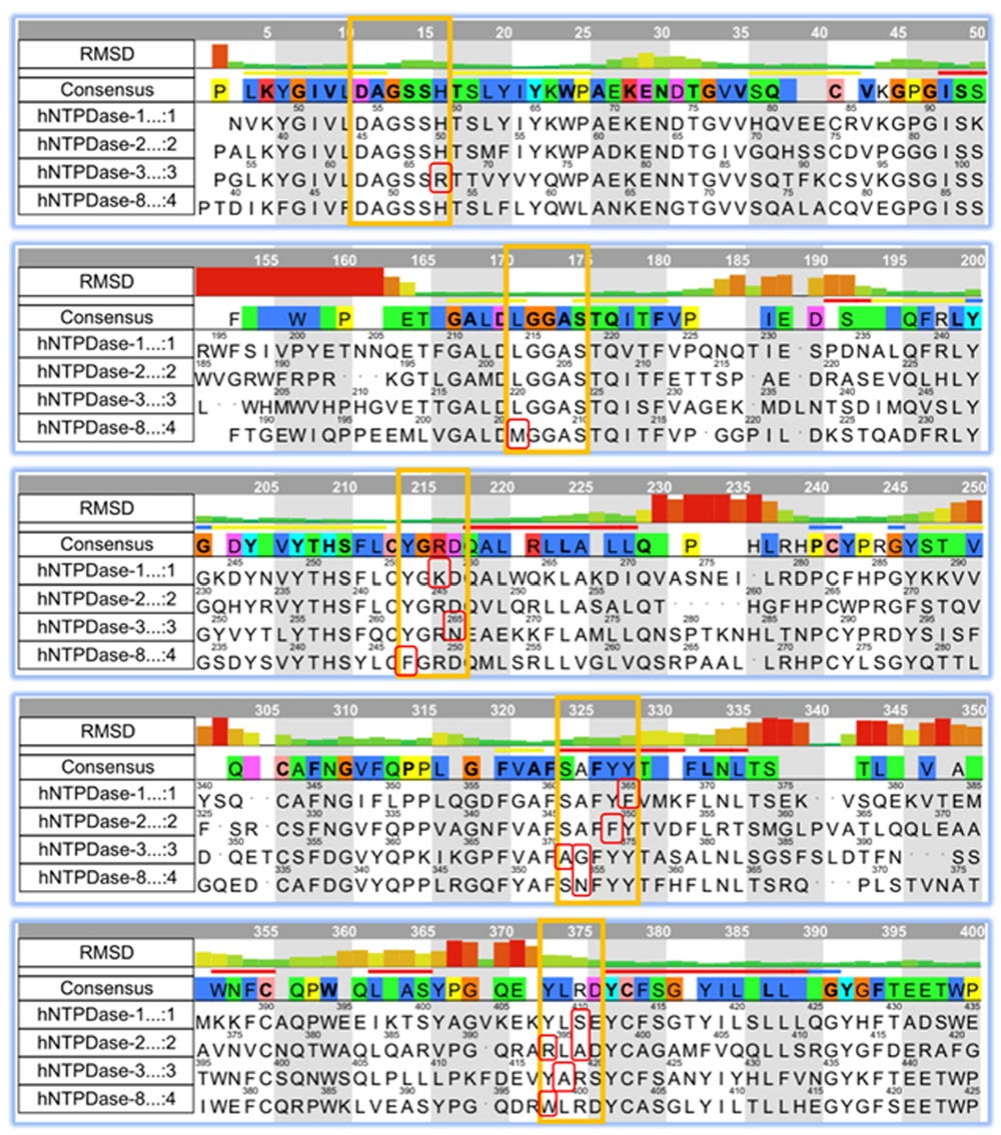
Sequence alignment of important regions of E-NTPDase isozymes. Residues delimited in orange color belongs to active site regions with conservative mutation highlighted in red.

From the superimposed structures of E-NTPDase isozymes, conservation of residues were determined. Most of the residues that makes up the active site were highly conserved except few conservative mutations where histidine is replaced with arginine, tyrosine with phenylalanine, lysine with arginine and aspartate with asparagine. All active site residues were found to deviate with very less magnitude from their homology model as compared to other parts of the protein. Figure 3 shows sequence alignment of active site region with their RMSD bar graphs.

### **B**inding Mode Analysis

#### Metal Geometry

As reported previously^6^, binding mode analysis also confirmed that the presence of divalent metal ion such as Ca^+2^ or Mg^+2^ ion inside active site is of crucial importance as it provides stability to the substrate molecule for subsequent hydrolysis. Previously reported crystal structure of rat ENTPDase-2 revealed that divalent metal ion forms octahedral geometry utilizing four water molecules and two oxygen atoms of α- and β-phosphate of Adenylyl-imidodiphosphate (AMP-PNP)^33,34^. In the course of docking study, ignoring metal ion in active site of the modelled proteins revealed very poor binding scores and odd poses of ligand molecules. However, with the consideration metal ion in the active site, the ligand molecules revealed very high binding affinity and uniformity of poses. Table 4 shows binding affinities of substrates inside E-NTPDase isozymes and Table 5 shows the comparative binding affinities of the standard inhibitor with and without metal ion consideration.

**Table 4:** Binding affinity of substrates inside E-NTPDase isozymes.

E-NTPDase Isozyme	Binding Free Energy ΔG (KJmol^−1^)			
	ADP	ATP	UDP	UTP
1	−25	−23	−12	−13
2	−16	−19	−17	−21
3	−15	−32	−18	−21
8	−22	−36	−36	−35

**Table 5:** Docking Scores of adenylyl-imidodiphosphate (AMP-PNP) inside ENTPDase isozymes.

E-NTPDase Isozyme	FlexX Docking Score	Binding Free Energy With Metal ion (KJmol^−1^)	Binding Free Energy Without Metal ion (KJmol^−1^)	Residues forming Interactions
1	−38.81	−28	−2	Ser57, Ser58, Thr131, Ala132, Ala217
2	−35.37	−14	−8	Ser48, Ser49, Gly47, Ala123, Gly203, Gly204, Tyr412, Tyr350
3	−54.11	−25	−3	Gly64, Ser65, Ser66, Thr139 Ala223 and Ser224.
8	−22.63	−21	−4	Gly50, Ser51, Ser52, His53 and Ser55

#### Substrate binding mode inside E-NTPDase1 isozyme

Substrate hydrolysis inside E-NTPDase1, revealed comparatively higher binding affinity of −25 KJmol^−1^ for ADP and −23 KJmol^−1^ for ATP. This difference in binding affinity can be related to the fact that ADP hydrolyze to AMP more readily as compared to its production by subsequent ATP hydrolysis. However, for UTP and UDP, higher binding affinity of −13 KJmol^−1^ was observed for UTP in contrast to lower binding affinity of −12 KJmol^−1^ for UDP. These observations strongly advocate the previously reported lower concentration of ADP and significantly higher concentration of UDP in reaction mixture as compared to ATP/UTP and AMP/UMP^16^.

**Figure 4:**
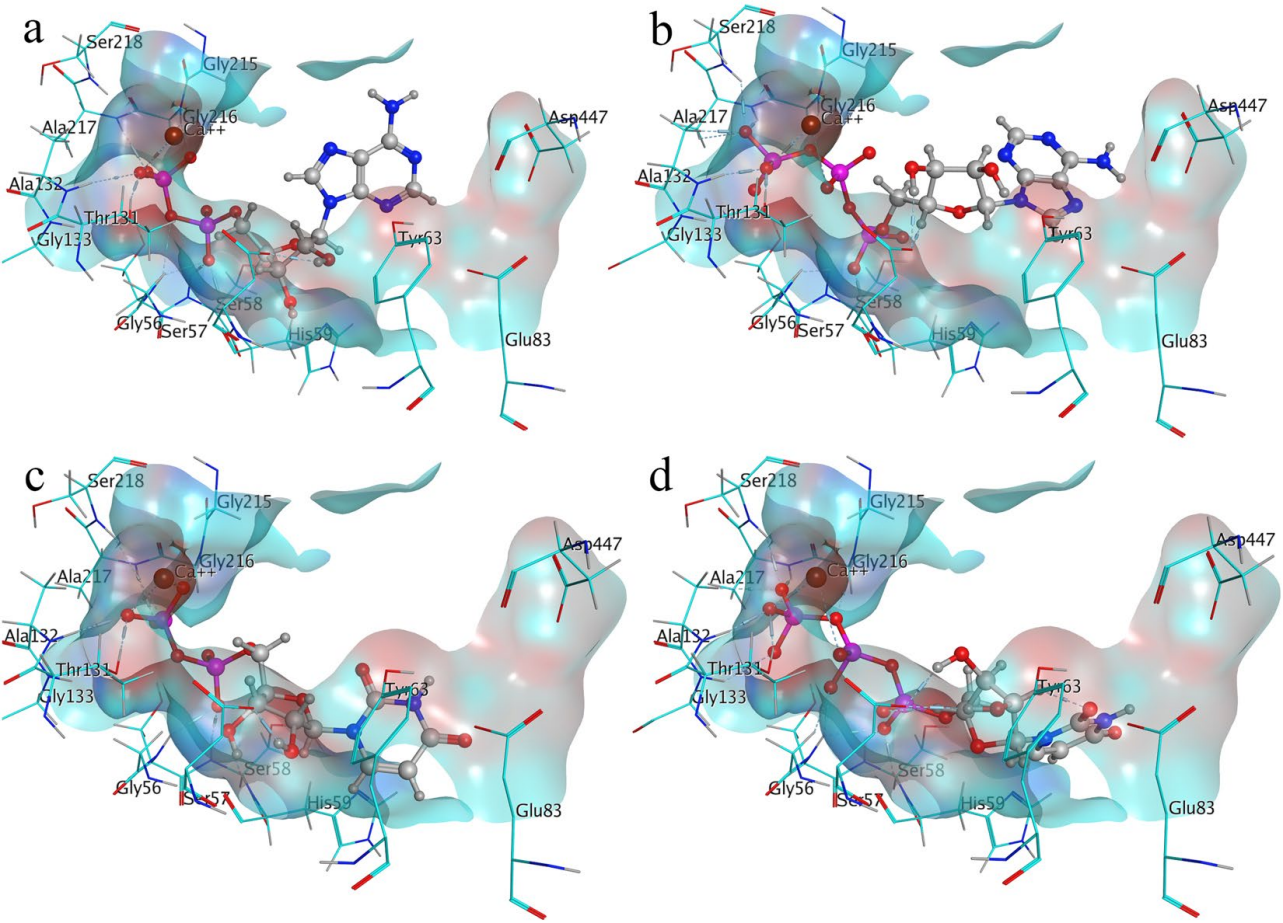
Binding mode of ADP (**a**), ATP (**b**), UDP (**c**) and UTP (**d**) inside human E-NTPDase1 (Dark blue color represents nitrogen, red is oxygen, pink color shows phosphorus, whereas, calcium is small brown sphere).

Binding study showed that phosphate group of substrates forms important hydrogen bonding interactions inside E-NTPDase isozymes, while nucleoside (adenine or uridine) group was found to be mainly involved in Pi-Pi interactions. The divalent metal ion plays a crucially important role of stabilizing the phosphate group by forming hydrogen bonding interactions with oxygen atoms of the two phosphate groups which undergoes hydrolysis. Amino acid residues Ser57, Ser58, Thr131, Ala132, Gly216 and Ala217 forms hydrogen bonding interactions with oxygen atoms of phosphates groups. In addition to hydrogen bonding, noticeable interactions are dipole dipole, showed by hydrogen atoms of amino acids and partially negative oxygen of the substrate (ADP, ATP, UDP and UTP). In addition to these, metal/ion interactions are also found between calcium and oxygen of the substrate. Figure 4 shows binding mode of substrates inside E-NTPDase1 active site.

#### Substrate binding mode inside E-NTPDase2 isozyme

E-NTPDase2 was found to have binding affinity of −19 KJmol^−1^ for ATP and −21 KJmol^−1^ in case of UTP. ADP and UDP showed −16 KJmol^−1^ and −17 KJmol^−1^ binding affinity inside E-NTPDase2, respectively. Because of higher binding affinity of triphosphonucleosides substrates (ATP and UTP) inside the enzyme active site, their rate of hydrolysis would be very high as compared to their diphosphonucleosides (ADP and UDP) leading to its accumulation in the reaction mixture as suggested in the previous study^17^.

**Figure 5:**
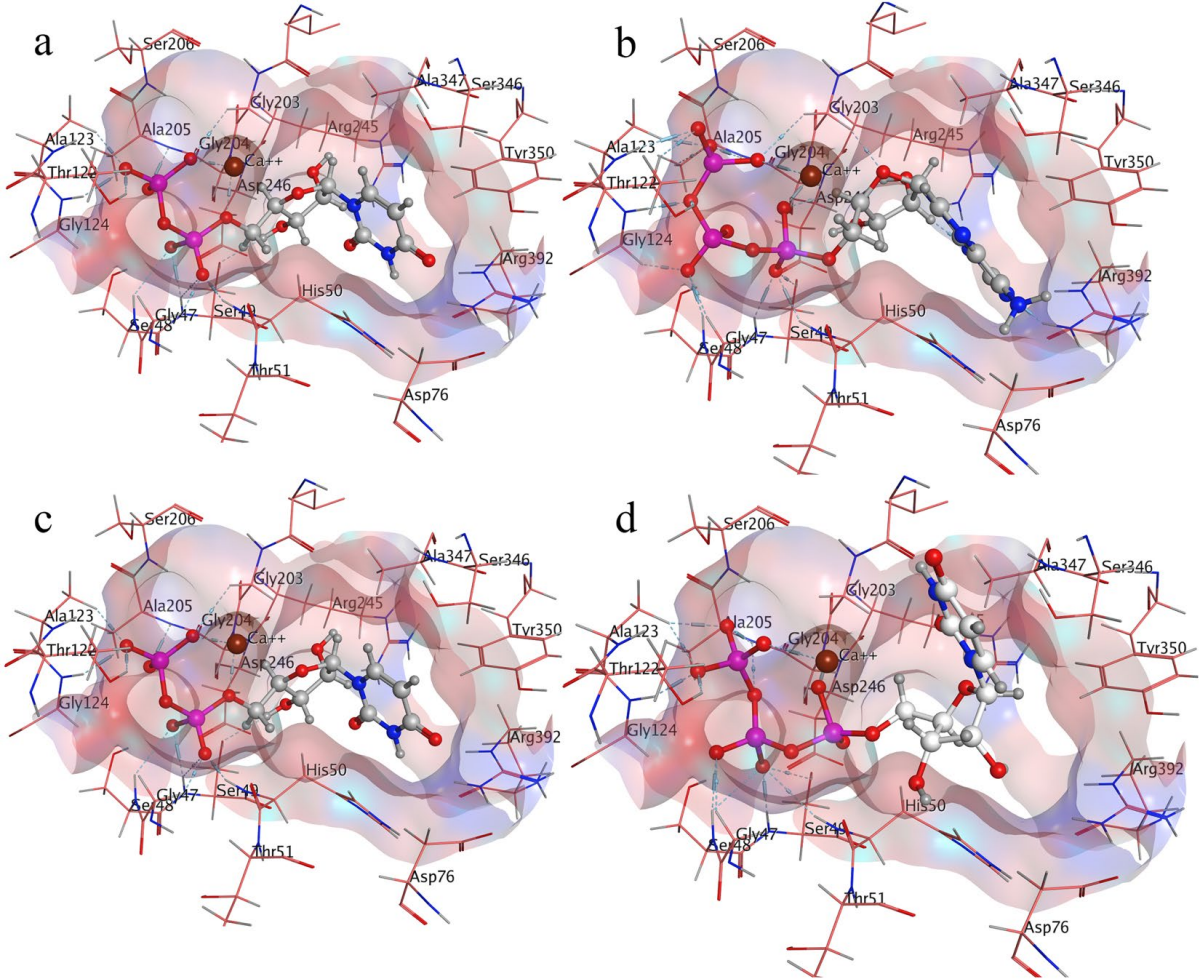
Binding mode of ADP (**a**), ATP (**b**), UDP (**c**) and UTP (**d**) inside human E-NTPDase2 (Dark blue color represents nitrogen, red is oxygen, pink color shows phosphorus, whereas, calcium is small brown sphere).

Inside active pocket of E-NTPDase2 divalent metal ion was found to form hydrogen bonding interactions with oxygen atoms of α- and β-phosphates groups of ADP and UDP and with oxygen atoms of α- and γ-phosphate of ATP and UTP. Amino acid residues Gly47, Ser48, Ser49, Thr122, Ala123, Gly203 and Gly204 forms hydrogen interactions with oxygen atoms of phosphate group. Moreover, dipole dipole interactions are noted by hydrogen atoms of amino acids and partially negative oxygen of the substrate. In addition to these, metal/ion interactions are also found between calcium and oxygen atom of the substrate (ADP, ATP, UDP and UTP). See Figure 5 for detailed binding poses of substrates.

#### Substrate binding mode inside E-NTPDase3 isozyme

**Figure 6:**
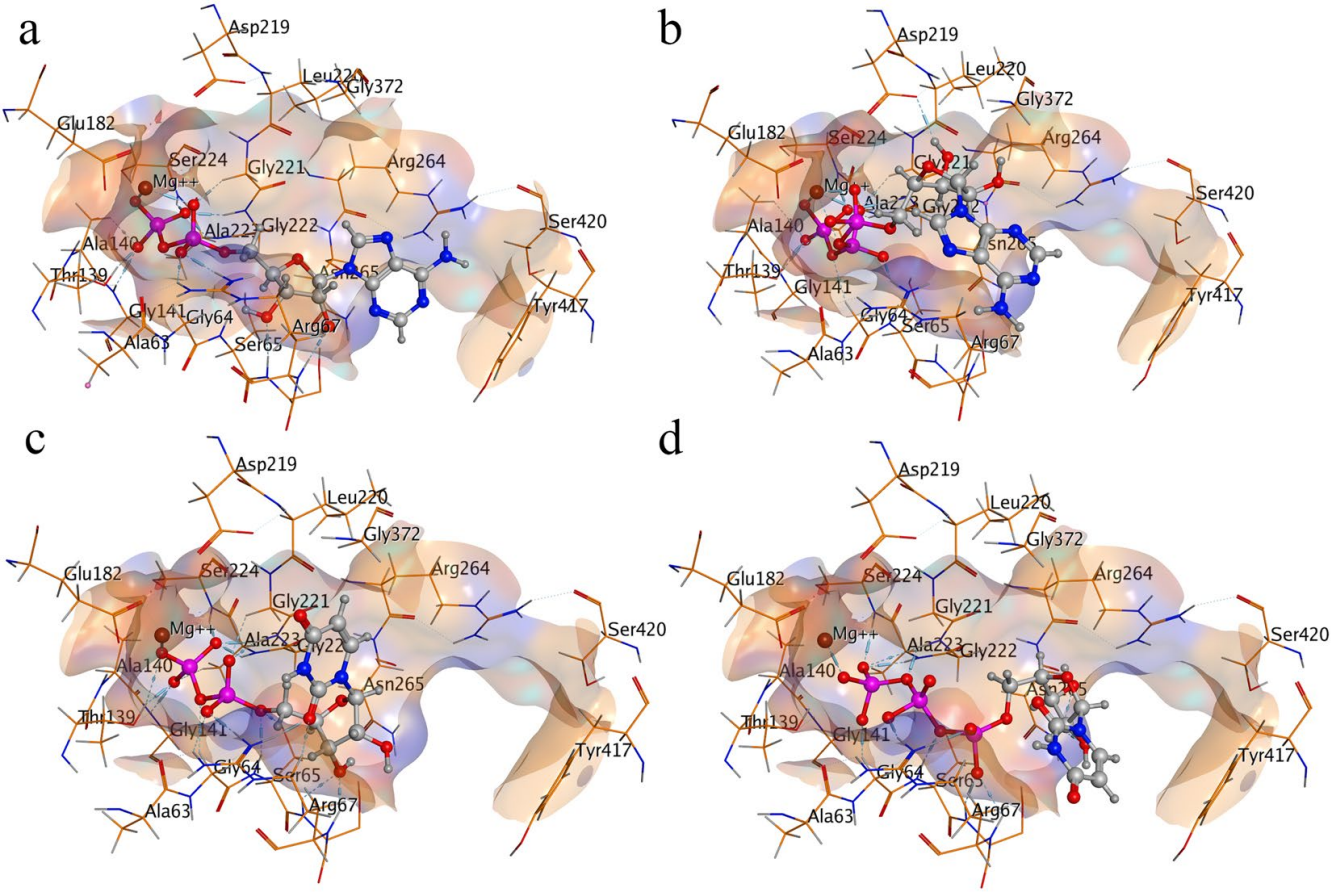
Binding mode of ADP (**a**), ATP (**b**), UDP (**c**) and UTP (**d**) inside human E-NTPDase3 (Dark blue color represents nitrogen, red is oxygen, pink color shows phosphorus, whereas, magnesium is small brown sphere).

Substrates hydrolysis inside E-NTPDase3 revealed similar pattern to that of E-NTPDase2 with higher binding affinity towards triphosphonucleosides as compared to diphosphonucleosides. Higher binding affinity of −32 KJmol^−1^ and −21 KJmol^−1^ was determined for ATP and UTP. For ADP and UDP showed considerably lower binding affinities of −15 KJmol^−1^ and −18 KJmol^−1^, respectively. Analogous to E-NTPDase2 binding mode, divalent metal ion formed similar hydrogen bonding interactions. Residues forming hydrogen bonding contacts with oxygen atoms of phosphate groups includes Gly64, Ser65, Arg67 and Thr139. Besides hydrogen bonding, noticeable interactions are dipole dipole, showed by hydrogen atoms of amino acids and oxygen atom of the substrate. In addition to these, metal/ion interactions are found between magnesium ion and oxygen of the substrate (ADP, ATP, UDP and UTP). Figure 6 shows binding poses of substrates inside E-NTPDase3 isozyme.

### Substrate binding mode inside E-NTPDase8 isozyme

**Figure 7:**
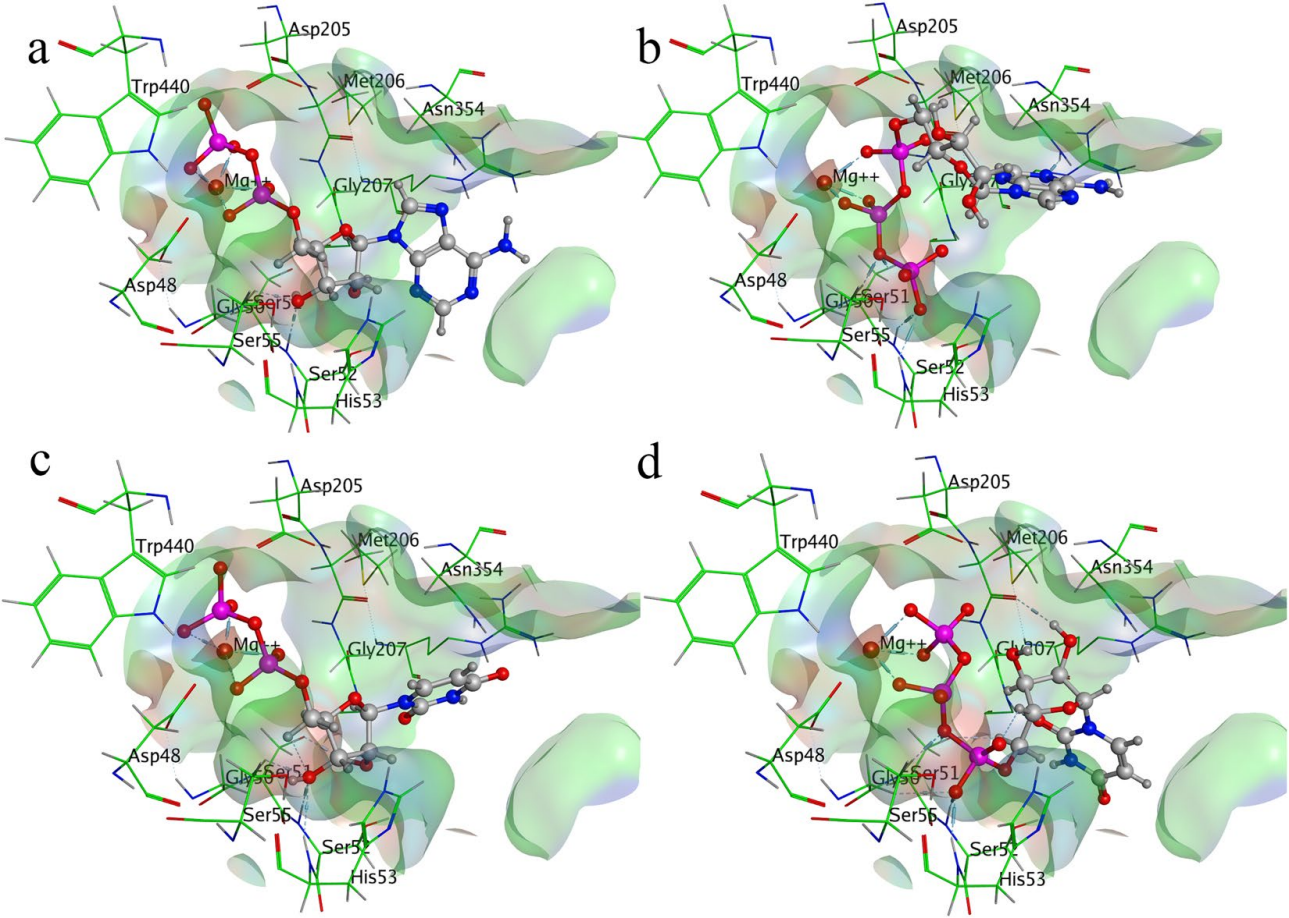
Binding mode of ADP (**a**), ATP (**b**), UDP (**c**) and UTP (**d**) inside human E-NTPDase8 (Dark blue color represents nitrogen, red is oxygen, pink color shows phosphorus, whereas, magnesium is small brown sphere).

Inside E-NTPDase8, UDP binds with higher binding affinity of −36 KJmol^−1^ as compared to ADP with −22 KJmol^−1^. Binding affinities in case of ATP and UTP was found to be −36 KJmol^−1^ and −35 KJmol^−1^, respectively. Binding pose analysis revealed similar pattern as observed for other surface located isozymes, divalent metal ion form hydrogen bonding interactions with oxygen atoms of α- and β- or β- and γ-phosphate of diphosphonucleosides or triphosphonucleosides, respectively. Residues Ser51, Ser52, His53 and Gly207 formed important hydrogen bonding interactions with oxygen atoms of phosphate groups. Along with hydrogen bonding, noticeable interactions are dipole dipole, showed by hydrogen atoms of amino acids and partially negative oxygen of the substrate (ADP, ATP, UDP and UTP). In addition to these, metal/ion interactions are also found between magnesium and oxygen of the substrate. Binding poses of substrate are shown in Figure 7.

#### Competitive Inhibitor - Adenylyl-imidodiphosphate binding mode inside E-NTPDase Isozymes and effect of metal ions on its binding affinity

**Figure 8:**
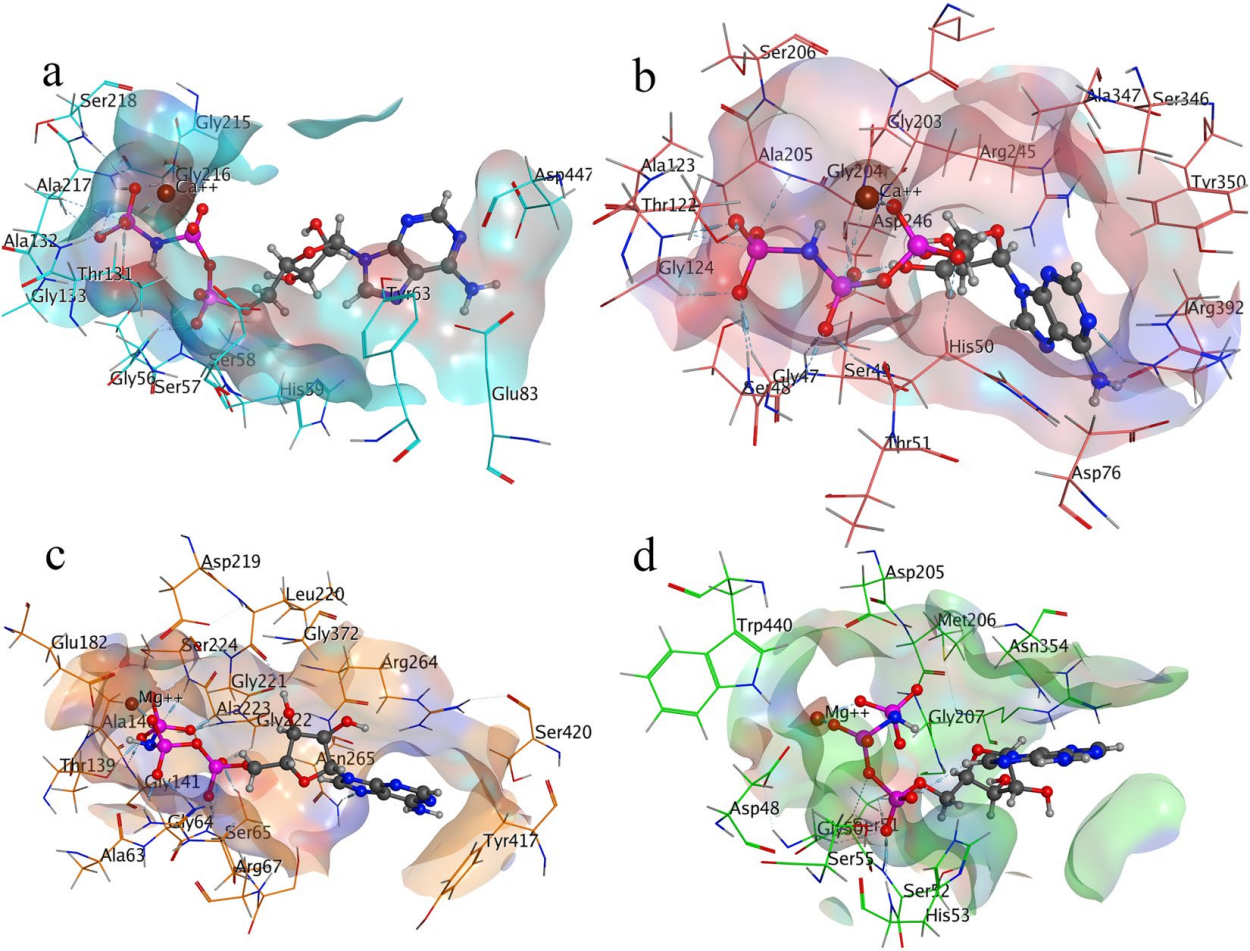
Putative binding mode of AMP-PNP inside E-NTPDase1 (**a**), E-NTPDase2 (**b**), E-NTPDase3 (**c**) and E-NTPDase8 (**d**) inside the active pocket (Dark blue color represents nitrogen, red is oxygen, pink color shows phosphorus, whereas, brown color small sphere is calcium in **a** and **b**, while, magnesium in **c** and **d**).

Competitive inhibitor, adenylyl-imidodiphosphate (AMP-PNP) was docked inside E-NTPDase isozymes with and without metal ion consideration inside the active site. It was observed that inside the active site of E-NTPDase isozymes, higher binding affinity and uniformity of docked poses were obtained as compared to docking inside isozymes without any metal ion consideration. Furthermore, the competitive inhibitor was found to form similar binding mode as that of triphosphonucleosides. The only difference in ATP and AMP-PNP is the presence of an imido group that render it resistant to hydrolysis. The key amino acid residues forming interactions are given in Table 5 and putative binding mode of AMP-PNP is shown in Figure 8.

E-NTPDase isozymes constructed by using homology modelling had been refined to the highest possible quality and can be used for subsequent *in-silico* studies. Binding mode analysis of the standard substrate molecules hallmarked the importance of divalent cations and the contributing amino acid residues in hydrolysis. Binding mode analysis of standard comparative inhibitor can serve as a guide to develop more specific inhibitors by exploiting the use of fragment and structure based drug design.

## Method

### Homology Modeling

Molecular Operating Environment^39^ was used to generate homology models of the cell surface located ecto-nucleotidases. The amino acid target sequences (UniProt ID P49961, Q9Y5L3, O75355 and Q5MY95) were obtained from UniProtKb-NCBI protein database. The corresponding template sequences were determined by using BLAST – Basic Local Alignment Search Tool of NCBI^43^. Identified template structures were then downloaded from RCSB protein data bank and fetched into software. Target sequences and the corresponding template structures were aligned using structure-oriented sequence alignment in MOE. Default parameters of the software were used for modeling of all proteins structures. Modeling of proteins were carried out by selecting parameters such as disabled C-terminal and N-terminal out-gaps and inclusion of the automatic detection of the disulfide bridges. Ten homology models were generated and the top ranking model was further refined and energy minimized by using Amber12:EHT force field and protonation was carried out using Protonate 3D tool of MOE^44^. Refined homology models were then superposed on their respective crystal structures to determine their closeness with their template structures in terms of their root mean square deviation (RMSD) values and conservation of amino acid residues especially at active site was determined. Stereochemical quality of the model was then assessed by Rampage Ramachandran plot analysis^45^.

### Molecular Dynamic Simulation

Using GROMACS (Groningen Machine for Chemical Simulation) software package, previously refined homology models of E-NTPDase 1, 2, 3 and 8 were subjected to 100 ns dynamic simulations in explicit water model^46,47^ using AMBER99Sb force field^48^. To represent water molecules, TIP3P model was used^49^. A 100 mM of Ca^+2^ and 200 mM of Cl^−1^ ion concentration was added to the proteins structures and the whole system was neutralized to pH 7. The protein structures were minimized to the point when the maximum force experienced by the system was in the order of 10^2^–10^3^ KJmol^−1^nm^−1^. Before running the production run, the protein system was equilibrated by running 100 ps of NVT (isothermal-isochoric) and NPT (isothermal-isobaric) ensemble. The simulation system was closely monitored to reach 300 K temperature and around 1 atmospheric pressure. Molecular dynamic simulations were then carried out in periodic cubic box with minimum distance of 1.0 nm between any atom of the protein and walls of the cubic box. Twin-range method, van der Waals and coulombs interactions were used to evaluate non-bonded interactions with cutoff range of 1.0 nm. Root mean square deviation (RMSD) of protein (backbone and side chains) as well as root mean square fluctuation (RMSF) of amino acid residues were plotted using XMGRACE v5.1.19^50^.

### Binding Mode analysis

In order to get insights into differential substrate hydrolysis carried out by E-NTPDase isozymes, molecular docking study of adenosine and uracil di- and triphosphates (ADP, ATP, UDP and UTP) was carried out. While AMP-PNP docking study was performed with an aim to determine inhibition mechanism of the competitive inhibitors. Chemical structures of the ligands were sketched and 3D clean in Marvin Sketch v15.11.30 from ChemAxon Ltd^51^.

To the refined isozymes structures, divalent metal ions such as Ca^+2^ and Mg^+2^ ions coordinates were added after realigning them with their respective template structures. To minimize steric hindrance or clashes around newly introduced metal ion, localized energy minimization of approximately 4 Å radius around divalent metal ion was performed.

FlexX utility embedded in LeadIT v2.20 software from BioSolveIT GmbH, Germany was utilized for docking studies^52^. The E-NTPDase isozymes were fetched and processed using ‘Load or Prepare Receptor’ utility. Automatic detection of binding cavities in LeadIT software also identified same binding sites as found in case of Site Finder of MOE. For all E-NTPDase isozymes the largest binding cavity with di-valent metal ion was used as site for docking studies. Hybrid enthalpy and entropy approach of docking was used with all other default parameters. Top ranking 50 poses for each ligand molecule were kept and validated further through HYDE visual affinity assessment which is a function of two parameters namely, hydrogen bonding affinity and dehydration energy estimation^53^. For each docked pose binding affinity (ΔG) was determined. Poses with lowest binding free energy values were considered as final poses.

## Electronic supplementary material

Supplementary Information

## References

[1] Muller CE (2002). P2-pyrimidinergic receptors and their ligands. Curr. Pharm. Des..

[2] North RA (2002). Molecular physiology of P2X receptors. Physiol. Rev..

[3] Lazarowski ER (2003). Molecular and biological properties of P2Y receptors. Curr. Top. Membr..

[4] Plesner L (1995). Ecto-ATPases: identities and functions. Int. Rev. Cytol..

[5] Robson SC, Sévigny J, Zimmermann H (2006). The E-NTPDase family of ectonucleotidases: structure function relationships and pathophysiological significance. Purinergic Signal..

[6] Zimmermann H (2001). Ectonucleotidases: some recent developments and a note on nomenclature. Drug Dev. Res..

[7] Bigonnesse F (2004). Cloning and Characterization of Mouse Nucleoside Triphosphate Diphosphohydrolase-8. Biochemistry.

[8] Handa M, Guidotti G (1996). Purification and cloning of a soluble ATP-diphosphohydrolase (apyrase) from potato tubers (Solanum tuberosum). Biochem. Biophys. Res. Commun..

[9] Vasconcelos EG (1996). Partial Purification and Immunohistochemical Localization of ATP Diphosphohydrolase from Schistosoma mansoni Immunological cross-reactivities with potato apyrase and Toxoplasma Gondii nucleoside triphosphate hydrolase. ‎J. Biol. Chem..

[10] Schulte am Esch J (1999). Structural elements and limited proteolysis of CD39 influence ATP diphosphohydrolase activity. Biochemistry.

[11] Smith TM, Kirley TL (1999). Site-directed mutagenesis of a human brain ecto-apyrase: evidence that the E-type ATPases are related to the actin/heat shock 70/sugar kinase superfamily. Biochemistry.

[12] Smith TM, Kirley TL (1999). Glycosylation is essential for functional expression of a human brain ecto-apyrase. Biochemistry.

[13] Smith TM, Lewis Carl SA, Kirley TL (1999). Mutagenesis of two conserved tryptophan residues of the E-type ATPases: inactivation and conversion of an ecto-apyrase to an ecto-NTPase. Biochemistry.

[14] Drosopoulos JH (2000). Site-directed mutagenesis of human endothelial cell ecto-ADPase/soluble CD39: requirement of glutamate 174 and serine 218 for enzyme activity and inhibition of platelet recruitment. Biochemistry.

[15] Grinthal A, Guidotti G (2000). Substitution of His59 converts CD39 apyrase into an ADPase in a quaternary structure dependent manner. Biochemistry.

[16] Kukulski F (2005). Comparative hydrolysis of P2 receptor agonists by NTPDases 1, 2, 3 and 8. Purinergic Signal..

[17] Sévigny J (2002). Differential catalytic properties and vascular topography of murine nucleoside triphosphate diphosphohydrolase 1 (NTPDase1) and NTPDase2 have implications for thromboregulation. Blood.

[18] Lecka, J., Fausther, M., Künzli, B. & Sévigny, J. Ticlopidine in its prodrug form is a selective inhibitor of human NTPDase1. *Mediat. Inflamm* (2014). 10.1155/2014/547480 PMC4144158 25180024

[19] Marcus AJ (2003). Metabolic control of excessive extracellular nucleotide accumulation by CD39/ecto-nucleotidase-1: implications for ischemic vascular diseases. J. Pharm. Exp. Ther..

[20] Lavoie EG (2010). Identification of the ectonucleotidases expressed in mouse, rat, and human Langerhans islets: potential role of NTPDase3 in insulin secretion. Am J. Physiol. Endocrinol. Metab..

[21] Cieślak M, Roszek K (2014). Purinergic signaling in the pancreas and the therapeutic potential of ecto-nucleotidases in diabetes. Acta Biochim. Pol..

[22] R-Candela J, Martin-Hernandez D, Castilla-Cortazar T (1963). Stimulation of insulin secretion *in vitro* by adenosine triphosphate. Nature.

[23] Petit P (1998). Evidence for two different types of P2 receptors stimulating insulin secretion from pancreatic B cell. Br. J. Pharmacol..

[24] Weir GC, Knowlton SD, Martin DB (1975). Nucleotide and nucleoside stimulation of glucagon secretion. Endocrinology.

[25] Ismail NA, El Denshary E (1977). & Montague, W. Adenosine and the regulation of insulin secretion by isolated rat islets of Langerhans. ‎Biochem. J..

[26] Munkonda MN (2007). Inhibition of human and mouse plasma membrane bound NTPDases by P2 receptor antagonists. Biochem Pharmacol..

[27] Westfall TD, Kennedy C, Sneddon P (1997). The ecto-ATPase inhibitor ARL 67156 enhances parasympathetic neurotransmission in the guinea-pig urinary bladder. Eur. J. Pharmacol..

[28] Drakulich DA, Spellmon C, Hexum TD (2004). Effect of the ecto-ATPase inhibitor, ARL 67156, on the bovine chromaffin cell response to ATP. Eur. J. Pharmacol..

[29] Gendron F-P (2000). Novel inhibitors of nucleoside triphosphate diphosphohydrolases: chemical synthesis and biochemical and pharmacological characterizations. ‎J. Med. Chem..

[30] Gendron F, Krugh O, Kong Q, Weisman G, Beaudoin A (2002). Purine signaling and potential new therapeutic approach: possible outcomes of NTPDase inhibition. Curr. Drug Targets..

[31] Zebisch M, Sträter N (2008). Structural insight into signal conversion and inactivation by NTPDase2 in purinergic signaling. Proc. Natl. Acad. Sci..

[32] Zebisch M, Krauss M, Schäfer P, Sträter N (2012). Crystallographic evidence for a domain motion in rat nucleoside triphosphate diphosphohydrolase (NTPDase) 1. J. Mol. Biol..

[33] Zebisch M, Krauss M, Schäfer P, Lauble P, Sträter N (2013). Crystallographic snapshots along the reaction pathway of nucleoside triphosphate diphosphohydrolases. Structure.

[34] Zebisch M, Baqi Y, Schäfer P, Müller CE, Sträter N (2014). Crystal structure of NTPDase2 in complex with the sulfoanthraquinone inhibitor PSB-071. J. Struct. Biol..

[35] Fan H, Mark AE (2004). Refinement of homology‐based protein structures by molecular dynamics simulation techniques. Protein Sci..

[36] Channar PA (2016). Isonicotinohydrazones as inhibitors of alkaline phosphatase and ecto‐5′‐nucleotidase. Chem. Biol. Drug Des..

[37] Khan I (2015). Investigation of quinoline-4-carboxylic acid as a highly potent scaffold for the development of alkaline phosphatase inhibitors: synthesis, SAR analysis and molecular modelling studies. RSC Adv..

[38] Ragno R (2007). Small molecule inhibitors of histone arginine methyltransferases: homology modeling, molecular docking, binding mode analysis, and biological evaluations. J. Med. Chem..

[39] Molecular Operating Environment v. 2015.09 (Chemical Computing Group Inc.).

[40] Karplus M, McCammon JA (2002). Molecular dynamics simulations of biomolecules. Nat. Struct. Mol. Biol..

[41] Karplus M, Petsko GA (1990). Molecular dynamics simulations in biology. Nature.

[42] Soga S, Shirai H, Kobori M, Hirayama N (2007). Use of amino acid composition to predict ligand-binding sites. J. Chem. Inf. Model..

[43] Altschul SF (1997). Gapped BLAST and PSI-BLAST: a new generation of protein database search programs. Nucleic Acids Res..

[44] Labute, P. Protonate 3D: assignment of macromolecular protonation state and geometry. Chemical Computing Group Inc (2007).

[45] Lovell SC (2003). Structure validation by Cα geometry: φ, ψ and Cβ deviation. Proteins: Struct., Funct., Bioinf..

[46] Berendsen HJ, van der Spoel D, van Drunen R (1995). GROMACS: a message-passing parallel molecular dynamics implementation. Comput. Phys. Comm..

[47] Abraham MJ (2015). GROMACS: High performance molecular simulations through multi-level parallelism from laptops to supercomputers. SoftwareX.

[48] Hornak V (2006). Comparison of multiple Amber force fields and development of improved protein backbone parameters. Proteins: Proteins: Struct. Funct., Bioinf..

[49] Jorgensen WL, Chandrasekhar J, Madura JD, Impey RW, Klein ML (1983). Comparison of simple potential functions for simulating liquid water. J. Chem. Phys..

[50] Turner, P. XMGRACE, Version 5.1. 19. Center for Coastal and Land-Margin Research, Oregon Graduate Institute of Science and Technology, Beaverton, OR (2005).

[51] MarvinSketch v. 15.11.30 (ChemAxon Ltd, 2015).

[52] LeadIT v. 2.1.8 (BioSolveIT GmbH, Germany, 2014).

[53] Schneider N, Lange G, Hindle S, Klein R, Rarey M (2013). A consistent description of HYdrogen bond and DEhydration energies in protein–ligand complexes: methods behind the HYDE scoring function. J. Comput. Aided Mol. Des..

