# Supplementary material for: Molecular dynamic simulations reveal structural insights into substrate and inhibitor binding modes and functionality of Ecto-Nucleoside Triphosphate Diphosphohydrolases
Source: Sci Rep. 2018 Feb 7;8:2581. doi: 10.1038/s41598-018-20971-4 (PMC5803232; doi:10.1038/s41598-018-20971-4)
Supplement: Supplementary file 1 — Supplementary Information [file 41598_2018_20971_MOESM1_ESM.pdf]

Supporting Information

Molecular dynamic simulations reveal structural insights into substrate and inhibitor binding modes and functionality of Ecto-Nucleoside Triphosphate Diphosphohydrolases

Jamshed Iqbal* and Syed Jawad Ali Shah

Centre for Advanced Drug Research, COMSATS Institute of Information Technology, Abbottabad, 22060, Pakistan.

Fig. S1 Complete Sequence Alignment of E-NTPDase Isozymes

Fig. S2a Ramachandran plot of E-NTPDase homology models.

Fig. S2b Ramachandran plot of E-NTPDase refined models after 100 ns MD simulation

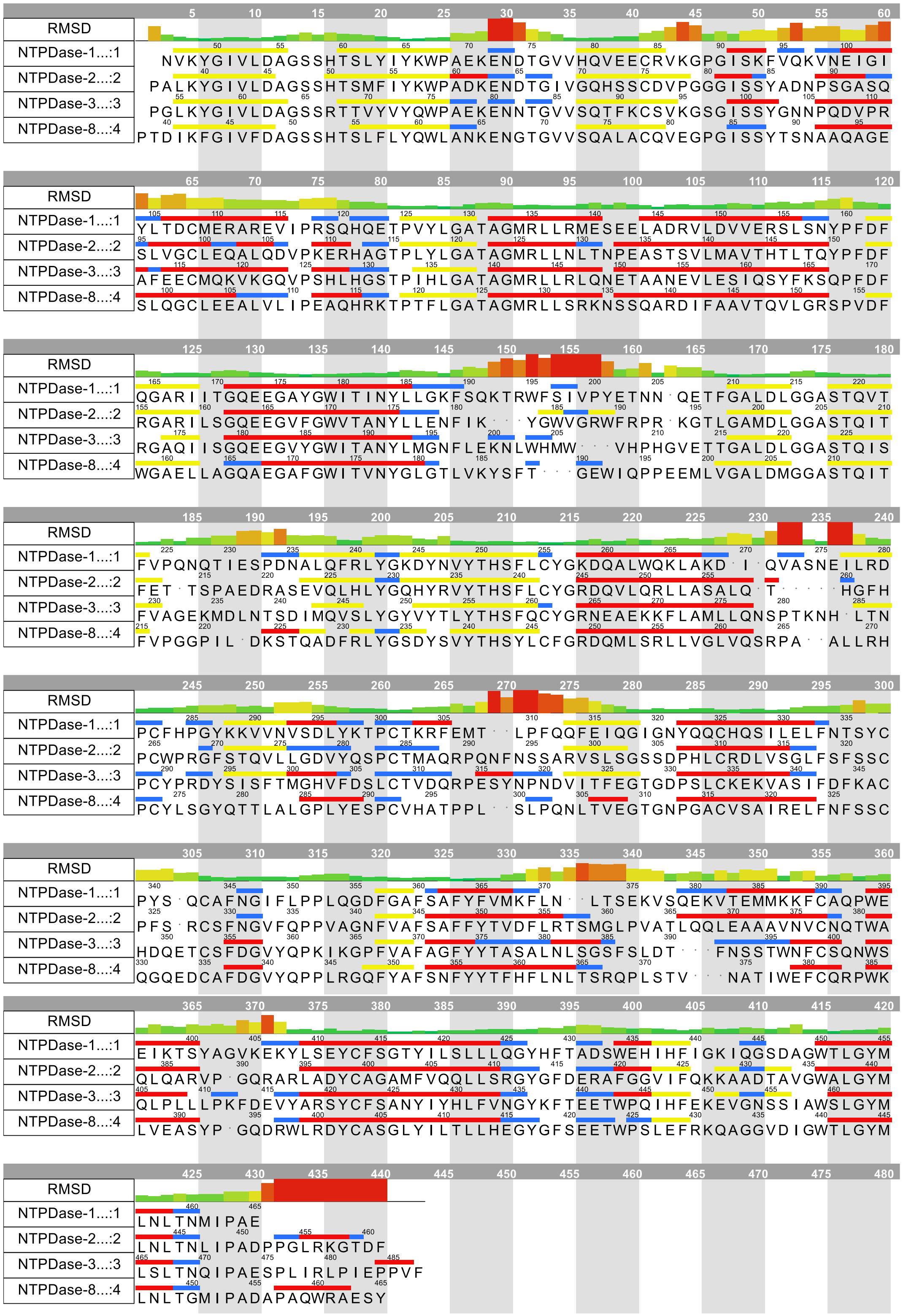

Fig. S1 Complete Sequence Alignment of E-NTPDase Isozymes with secondary structure details. (Red color bar shows α-helix, yellow shows β-pleated sheets and blue shows turns)

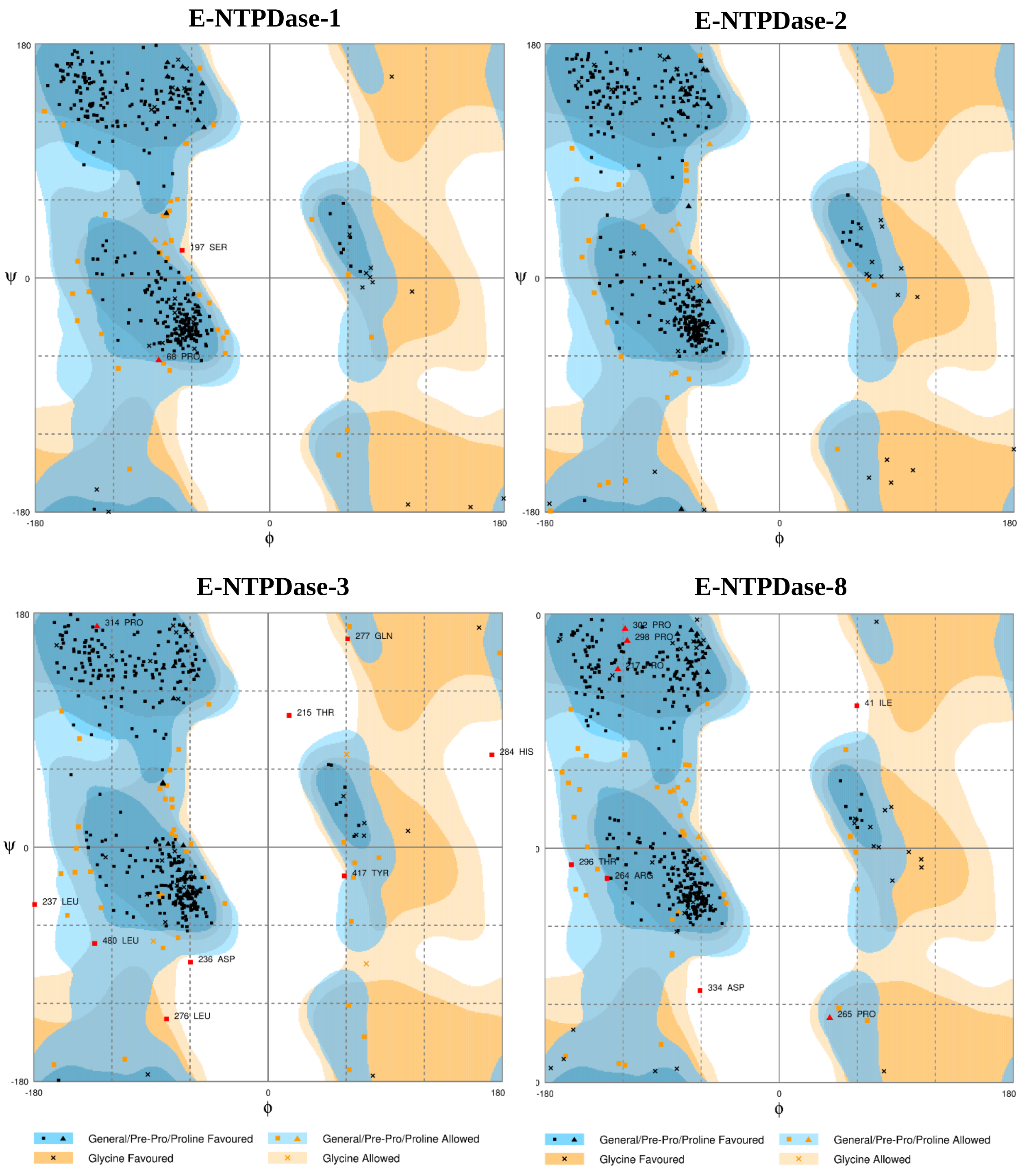

Fig. S2a Ramachandran plot of E-NTPDases homology models.

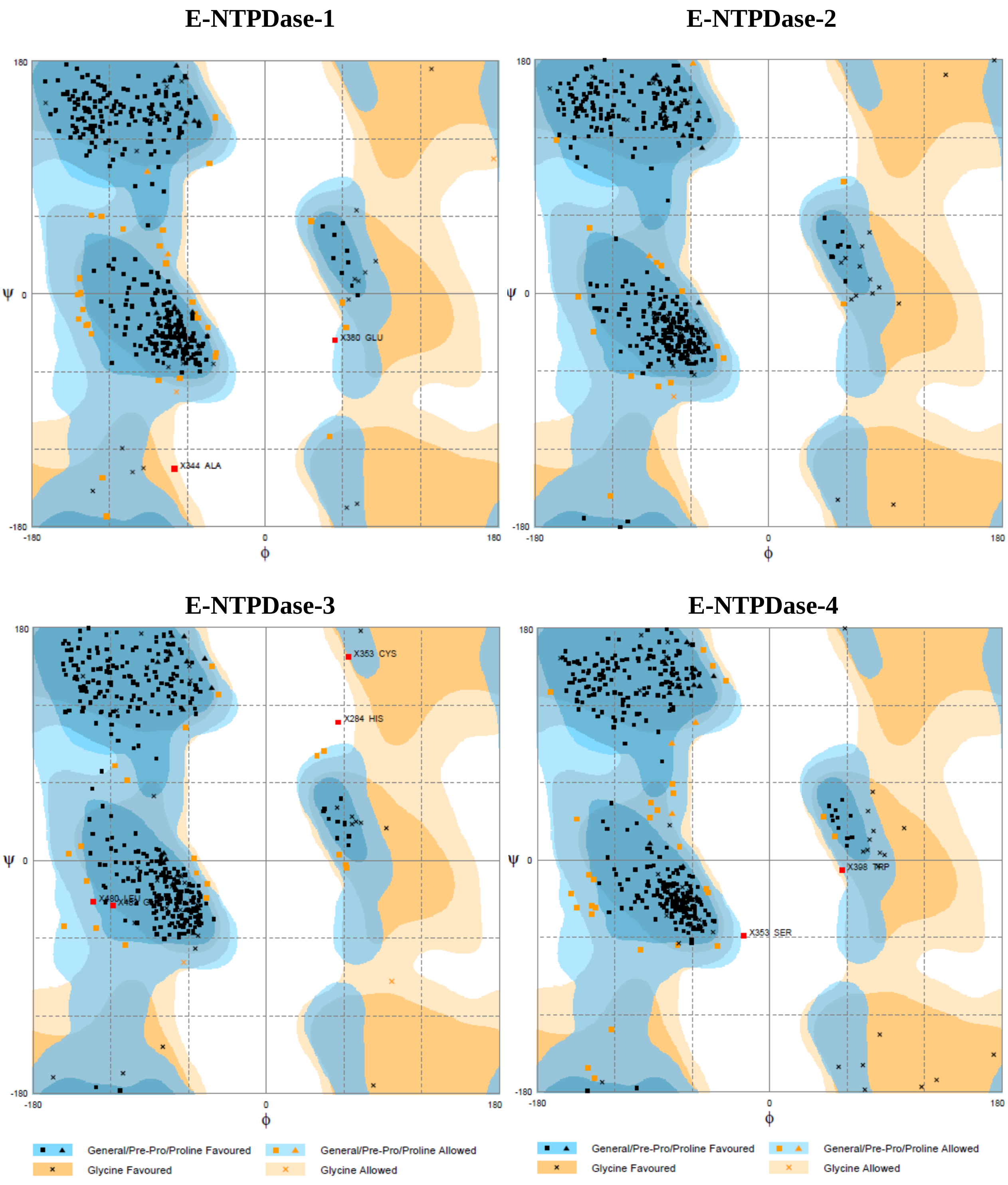

Fig. S2b Ramachandran plot of E-NTPDases refined models after 100 ns MD simulation
